# Xylanase and Direct-Fed Microbials (DFM) Potential for Improvement of Live Performance, Energy Digestibility, and Reduction of Environmental Microbial Load of Broilers

**DOI:** 10.3389/fvets.2020.606415

**Published:** 2020-12-07

**Authors:** Basheer Nusairat, Jeng-Jie Wang

**Affiliations:** ^1^Department of Animal Production, College of Agriculture, Jordan University of Science and Technology, Irbid, Jordan; ^2^BioResource International, Inc., Durham, NC, United States

**Keywords:** xylanase, *Bacillus* spp., DFM, antibiotic-free, energy digestibility, broiler

## Abstract

The challenge of identifying alternatives to subtherapeutic levels of antibiotic growth promoters (AGP) in animal feed has led to increased interest in feed additives such as exogenous enzymes and direct-fed microbials (DFM). Six corn soy-based dietary treatments were designed to investigate the effect of high-efficiency xylanase alone, *Bacillus* spp. probiotics alone, and their combination vs. a commonly used antibiotic growth promoter (bacitracin methylene disalicylate; BMD) on live performance and environmental *Clostridium perfringens* load of broiler chickens with eight replicate pens per treatment. Diets were as follows: standard diet (positive control; PC); 130 kcal/kg reduced-energy diet (negative control; NC); NC with xylanase (NC + Xy); NC with probiotics (NC + Pro); NC with xylanase and probiotics mix (NC + XyPro); and NC with BMD (NC + BMD). Data were analyzed as one-way ANOVA. At 35 and 42 days, birds fed with NC + XyPro and NC + BMD were heavier (*P* < 0.05) than birds fed with NC. Improvement in feed conversion ratio (FCR) (*P* = 0.0001) was observed from 1 to 42 days by ~3 points in both NC + XyPro and NC + BMD compared to NC. The NC + XyPro reduced lesion scores by 66% compared to PC and NC. Litter *C. perfringens* cell count was reduced by ~16% with supplementation of XyPro or BMD. It can be concluded that a blend of xylanase (10 XU/g feed) and *Bacillus* spp. [1 × 10^5^ colony forming units (CFU)/g feed] can be used as an alternative to AGP in low-energy broiler diets.

## Introduction

Poultry producers face challenges due to the ban on antibiotic growth promoter (AGP) use in feed. Europe has already implemented AGP-free production, and the US is currently working toward full implementation. More pressure has also been exerted by the consumer demand for AGP-free poultry meat at an affordable price. Furthermore, with human health being closely connected to animal health and the surrounding environment (one health concept), it becomes necessary for producers to seek alternatives that will comply with AGP-free rules and maintain profit without negatively impacting gut health, poultry performance, or the environment.

The earliest findings on using exogenous enzymes in poultry were reported by Clickner and Follwell (1), using Proto-Zyme enzyme from *Aspergillus oryzae*, leading to improved pullet performance. After that, exogenous enzymes were incorporated in poultry feed to reduce environmental pollution (2). Since then, enzymes targeting various substrates have become commercially available to improve feed and feed byproduct utilization by the animal, increase nutrient digestibility, and enhance performance (3, 4). A group of these exogenous enzymes targets non-starch polysaccharides (NSPs), which comprise ~90% of the plant cell wall (5) and are broadly classified into three categories: cellulose, non-cellulosic polymers, and pectic polysaccharides (6). Xylanases break down components present in the cell wall of cereal grains (7, 8), leading to a reduction in cell wall integrity, the release of trapped nutrients, as well as modifying intestinal microbial communities due to the prebiotic-like effect (9) exerted by oligosaccharide units resulting from hydrolysis, and have been reported to increase fat and starch digestibility (10). Xylanases are broadly classified into β-1,4-endoxylanase, which hydrolyze the β-(1–4)-linked xylan backbone to xylooligosaccharides in the arabinoxylans (non-cellulosic polymers) (11), and β-xylosidase that breaks down the xylooligomers to monomer xylose units (12, 13).

Xylanase allows for a better utilization of dietary energy, which has both economic value for poultry producers as well as environmental benefits (14, 15). Less amount of feed is required to produce meat and eggs, and fewer excess nutrients are excreted, thus reducing nutrients available for pathogen proliferation in the hind gut and in the litter (16). The utilization of low-energy diets to demonstrate the potential of xylanase has been adopted by multiple researchers (17, 18). Saleh et al. (18) showed that supplementation of xylanase and arabinofuranosidase to diets formulated with 90 kcal/kg less apparent metabolizable energy (AME) than a standard diet improved growth performance, nutrient digestibility, and immune response of broilers. The ability of xylanase to improve utilization of energy as a result of fiber hydrolysis and release of trapped nutrients could potentially allow for feeding diets with lower energy content thus reducing cost of production.

Another approach that can be implemented as an alternative to AGP is incorporating direct-fed microbials (DFM) in poultry feed (19). Supplementation of DFM in feed can be dated back to the 1970s (19). Different types of microorganisms have been used in animal production as DFM; the most common categories are bacterial, fungal, or a combination of both (20). Several researchers have shown the beneficial effect of DFM on broiler performance (21–23) and the modulation of intestinal microflora and pathogen inhibition (21–23). The primary function of DFM is enhancing gut microbiota stability by preventing the growth of pathogenic microorganisms such as *Salmonella* and *Campylobacter* (24, 25), improving immune functions in the intestines (20, 24), and ultimately benefiting livestock performance (26, 27). In order to exert these functions, a DFM product is required to be non-pathogenic, capable of surviving the hygienic parameters used during feed processing, and endure the harsh environment of the digestive system in order to reach the targeted area and deliver beneficial effects.

The combination of xylanase and DFM could provide both prebiotic (resulting from xylooligomer breakdown) and probiotic effects simultaneously. Reported research demonstrated that such combination could improve broiler performance and gut health and reduce pathogen load (28) through a synergistic effect, thus providing a promising successful alternative to AGP. It is hypothesized that this combination, when supplemented to a low-energy diet, could enhance the growth performance and gut health of broilers (28). Therefore, this study's objective was to evaluate the efficacy of a combination of high-efficiency xylanase blended with multi-strain *Bacillus* probiotic on live performance, gut lesions, and environmental *Clostridium perfringens* load of broiler chickens fed low-energy corn soy diets and reared under typical broiler production conditions when compared to an AGP.

## Materials and Methods

Animal care practices conformed to the Guide for the Care and Use of Agricultural Animals in Agricultural Research and Teaching (29).

### Experimental Design

A total of 2,496 Ross 708 mixed-sex 1 day-old broilers were obtained from a commercial hatchery and randomly placed in floor pens (305 cm × 152 cm) with eight replicate pens per treatment, each containing 52 chicks and raised to 42 days of age under typical US broiler production conditions in a completely randomized design with six experimental treatments. Chicks were reared on used litter topped with fresh pine wood shavings and given *ad libitum* access to feed and water. The lighting program included continuous light at an intensity of more than 3 foot-candles (fc) for the first week and then dimmed to 1 fc for the remainder of the trial.

### Experimental Diets

Six dietary treatments were designed to evaluate the effect of xylanase, multi-strain *Bacillus* spp. probiotics and their combination, as well as a commonly used antibiotic growth promoter, bacitracin methylene disalicylate (BMD). The diets (Table 1) were as follows: standard corn soybean meal broiler diet (positive control; PC), reduced-energy diet formulated at 130 kcal/kg lower than PC (negative control; NC), NC with xylanase (NC + Xy), NC with multi-strain *Bacillus* spp. probiotics (NC + Pro), NC with xylanase and probiotics mix (NC + XyPro), and NC with BMD (NC + BMD). To achieve the energy reduction, corn was partially replaced with soybean hulls in NC and NC-related diets, which also resulted in increased fiber content in these diets. However, the influence of fiber content was not evaluated directly. The NC and other experimental diets formulated with the enzyme and probiotic had similar fiber content, and therefore, it is not considered a confounding factor. The xylanase alone was added at the rate of 100 g/MT, which provided 10 XU of endo-β-1, 4-xylanase per gram of feed, and the probiotics alone was added at the rate of 100 g/MT, which provided 1 × 10^5^ colony forming units (CFU) of multi-strain *Bacillus* spp. per gram of feed. The xylanase and probiotic mix was added at the rate of 100 g/MT of feed, which provided 10 XU of endo-β-1, 4-xylanase and 1 × 10^5^ CFU of multi-strain *Bacillus* spp. per gram of feed. Both xylanase activity and *Bacillus* spp. enumeration were confirmed by analyzing the feed samples. The fixed inclusion rate of 100 g/MT was achieved by adding limestone as a carrier. The BMD was added at the rate of 50 g/MT. Diets were formulated to either meet or exceed nutrient requirements of broilers (30). Birds were fed mash starter (days 1 to 21), grower (days 22 to 35), and finisher diets (days 36 to 42).

**Table 1:** Composition and nutrient content of experimental diets.

	**Starter (1–21 days)**		**Grower (22–35 days)**		**Finisher (36–42 days)**	
	**Standard**	**Reduced energy**	**Standard**	**Reduced energy**	**Standard**	**Reduced energy**
**Ingredient (%)**						
Corn	60.77	56.42	67.37	63.02	71.55	67.76
Soybean meal 48%	28.12	27.74	23.31	22.93	14.89	20.92
Poultry meal	5.00	5.00	5.00	5.00	8.24	5.00
Poultry fat	0.05	0.05	0.05	0.05	0.05	0.05
DL-methionine	0.17	0.18	0.13	0.14	0.02	0.06
Salt	0.48	0.48	0.43	0.43	0.37	0.38
Lysine	0.14	0.16	0.15	0.17	0.18	0.08
Limestone	1.5	1.4	1.3	1.2	1.1	1.2
Dicalcium phosphate	2.0	2.0	1.7	1.7	1.6	1.6
Vitamin and mineral premix^a^	0.5	0.5	0.5	0.5	0.5	0.5
Soybean hulls	1.3	6.0	0.1	4.8	1.5	2.5
**Calculated nutrients (%)**						
Crude protein	22	22	20	20	19	19
Crude fat	1.44	1.39	1.87	1.84	1.81	2.12
Crude fiber	2.69	4.59	2.38	4.93	4.17	3.53
Ash	7.84	8.81	7.46	7.55	8.77	7.31
Calcium	1.05	1.05	0.9	0.9	0.85	0.85
Available phosphate	0.512	0.512	0.45	0.45	0.42	0.42
Sodium	0.22	0.22	0.2	0.2	0.18	0.18
Dig lysine	1.28	1.28	1.15	1.15	1.02	1.02
Dig methionine + cysteine	0.947	0.947	0.851	0.851	0.755	0.755
Metabolizable energy (kcal/kg)	2,998	2,868	3,100	2,970	3,199	3,069

a *Vitamin and trace mineral premix supplied the following per kilogram of diet: 5,512 IU vitamin A, 1,852 IU vitamin D3, 11 IU vitamin E, 0.06 mg vitamin B12, 0.23 mg biotin, 1.87 mg menadione (K3), 0.44 mg thiamine, 3.75 mg riboflavin, 5.95 mg d-pantothenic acid, 1.32 mg vitamin B6, 34.17 mg niacin, and 0.22 mg folic acid; mineral premix supplied the following per kilogram of diet: manganese: 120 mg, zinc: 120 mg, iron: 80 mg, copper: 10 mg, iodine, 2.5 mg, and cobalt, 1 mg*.

### Data Collection

#### Live Performance

Birds and feed were weighed at placement and at 21, 35, and 42 days for live performance measurements. Mortality was recorded as it occurred. Measurements were used for determining body weight (BW), body weight gain (BWG), feed consumption (FC), feed conversion ratio (FCR) adjusted for mortality, BW coefficient of variation (CV; flock uniformity), and percent mortality. The flock uniformity was based on the coefficients of variation based on individually measured BWs and how each BW deviated from the mean BW of each pen.

#### Environmental Pathogen Load

Counts of *Clostridium perfringens* (*C. perfringens*) were enumerated from litter as an indicator of environmental pathogen load. *C. perfringens* CFUs per gram of pen litter was measured prior to placement and at 21 and 42 days of age per FDA BAM, chapter 16 (31). Briefly, 25 g of litter was homogenized in 225 ml of peptone diluent (0.1% peptone), then 10-fold dilutions of each sample were prepared up to 10^9^. One (1) milliliter of each dilution was placed on Tryptose Sulfite Cycloserine Agar plates and incubated under anaerobic conditions at ~35°C for ~24 h. Plates were then removed from the incubator and total viable *C. perfringens* colonies were counted using dilution plates with ~20–200 CFUs. Samples were analyzed in quadruplicate.

#### Apparent Metabolizable Energy Digestibility

On days 19 and 40, four birds per pen (two males and two females) were randomly selected and moved to raised wire cages. Birds were fasted for 6 h followed by feeding of the respective diets until days 21 and 42, respectively. Feed consumption was measured per cage. All excreta was collected during the feeding period as well as during the 12 h after feed removal (post-feeding portion). Excreta samples were pooled per treatment then dried, processed, and analyzed for dry matter, gross energy, and nitrogen. Feed consumed was also analyzed for dry matter, gross energy, and nitrogen. The following calculations were used to determine apparent metabolizable energy (AME) and nitrogen corrected (AMEn):

$$\begin{array}{l} \text{AMEn}=[\text{FC}\times \text{GEfeed})-(\text{DMfecal}\times \text{GEfecal})-(\text{NR}\times 8.73)]/\text{FC} \end{array}$$

Where, FC = feed consumed, GEfeed = gross energy of feed, DMfecal = fecal dry matter, GEfecal = gross energy of feces, and NR = nitrogen retention, where NR = (FC × feed nitrogen) – (DMfecal × fecal nitrogen).

### Statistical Methods

Data were analyzed as one-way ANOVA in a completely randomized design (CRD) with six dietary treatments and eight replicate pens per dietary treatment. The general linear model of Statistical Analysis System (SAS, 2017) was employed. Means were separated by LSMEANS. Superscripts were determined based on PDIFF values. Live performance data were analyzed using pen as the experimental unit, while individual birds were considered the experimental units for microbial load and lesion scores with 32 birds per dietary treatment. Means were considered significantly different at a set *P* ≤ 0.05.

## Results and Discussion

### Live Performance

Live performance results are shown in Table 2 (FC, BW, BWG, FCR, CV of BW, and mortality). There were no significant differences for FC among treatments. However, differences in BW (data not shown) and BWG were detected starting at 21 days with a tendency (*P* = 0.06) for birds consuming NC + XyPro and NC + BMD to be heavier than birds on NC with NC + Xy and NC + Pro birds being intermediate. These trends became highly significant (*P* = 0.0001) at days 35 and 42 for BW and from 1 to 42 days (*P* = 0.0001) for BWG. Birds consuming NC + XyPro and NC + BMD gained ~2% more weight than NC, while NC + Xy gained 1% more weight, and NC + Pro gained ~0.8% more weight from 1 to 42 days. Supplementing either XyPro or BMD improved FCR compared to NC from 1 to 21 days (*P* = 0.0001) and 1 to 42 days (*P* = 0.004) by ~3 points in both NC + XyPro and NC + BMD compared to NC. Improvements in live performance observed in this study were achieved by the addition of xylanase and DFM blend to broilers in a mild microbially-challenged environment. Results (BWG and FCR) at 35 and 42 days are in agreement with the findings of de Oliveira et al. (32) when DFM were fed to broilers under enteric pathogen-challenged environment. Vandeplas et al. (33) also reported an improvement in growth rate when a combination of *Lactobacillus plantarum* and xylanase was supplemented to broilers infected with *Salmonella typhimurium*. Furthermore, birds were nutritionally challenged by reducing energy level in the diets (130 kcal/kg), which was achieved by the inclusion of soybean hulls, thus increasing fiber content. These observations were consistent with previous findings by Harrington et al. (34), who reported that broilers supplemented with *B. subtilis* had an improved final BW and FCR when fed reduced ME diets, while Williams et al. (16) reported improved FCR when xylanase was added to reduced energy diets (−132 kcal/kg).

**Table 2:** Least-square means for feed consumption (FC), body weight gain (BWG), feed conversion ratio (FCR), mortality, and BW coefficient of variation for broilers raised to 42 days.

	**Treatments**						**SE**	***P*-value**
**Age period (days)**	**PC**	**NC**	**NC + Xy**	**NC + Pro**	**NC + XyPro**	**NC + BMD**		
**FC (g/bird)**								
1–21	1,042	1,040	1,044	1,042	1,039	1,043	9	0.99
22–35	2,368	2,374	2,364	2,366	2,358	2,367	25	0.98
36–42	1,329	1,341	1,342	1,351	1,344	1,337	41	0.99
1–42	4,739	4,754	4,750	4,758	4,741	4,747	35	0.98
**BWG (g/bird)**								
1–21	806	777	786	784	792	795	7	0.06
22–35	1,326	1,280	1,291	1,290	1,303	1,311	11	0.07
36–42	697	673	687	678	693	688	17	0.94
1–42	2,829^a^	2,730^d^	2,764^bcd^	2,753^cd^	2,787^bc^	2,794^ab^	13	0.0001
**FCR (g:g)**								
1–21	1.291^a^	1.336^d^	1.322^bcd^	1.326^cd^	1.313^bc^	1.308^b^	0.005	0.0001
22–35	1.795	1.855	1.836	1.838	1.820	1.813	0.015	0.11
36–42	1.751	1.810	1.791	1.806	1.778	1.771	0.045	0.94
1–42	1.677^a^	1.736^c^	1.717^bc^	1.722^bc^	1.702^ab^	1.697^ab^	0.01	0.004
**Mortality (%)**								
1–21	1.44	1.68	1.68	1.44	1.44	1.68	0.67	0.99
22–35	0.12	0.66	0.40	0.38	0.38	0.14	0.23	0.58
36–42	0.0	0.26	0.0	0.52	0.26	0.26	0.28	0.78
1–42	1.56	2.60	2.08	2.34	2.08	2.08	0.79	0.96
**Flock uniformity (%)**								
21	13.7	14.3	13.7	13.9	13.5	13.9	0.4	0.63
35	8.5	9.1	9.2	9.0	9.1	8.6	0.2	0.18
42	9.1	9.3	9.4	9.2	9.3	9.4	0.2	0.90

a−d *Means in a row within each variable with no common superscript differ significantly (P ≤ 0.05)*.

*PC, positive control; NC, negative control with ME 130 kcal/kg lower than PC; NC + Xy, NC with xylanase; NC + Pro, NC with probiotic; NC + XyPro, NC with xylanase and probiotic; NC + BMD, NC with BMD; SE, standard error for n = 8 pens*.

Mortality results are presented in Table 2. Generally, the overall reported mortality from 1 to 42 days was low and was not affected by treatments. Individual BW data were analyzed for the coefficient of variation (CV) calculation as an indicator of flock uniformity at days 21, 35, and 42. Acceptable uniformity is indicated by a CV (percentage values) for BW <10%. Flock uniformity percentage at 21 days was more than 10%, which could be due to this being a straight-run flock (including both males and females); during the first 21 days, males and females differ in target organ nutrient deposition; males give priority for muscle accretion, mainly breast muscle, while females give priorities for feathering leading to a fully-feathered females by 21 days, whereas males become fully feathered by 35 days of age (35). By that time, fully-feathered females would have started depositing nutrients for muscle growth leading to overall improved flock uniformity seen at 35 and 42 days, which was similar to other researchers (32).

Xylanase exerts its effect mainly through reducing digesta viscosity; this is more profound when dietary fiber content, mainly NSPs, is increased. The soluble portion of NSPs (mainly arabinoxylans) impairs nutrient digestion and absorption due to nutrient encapsulation (36), thus reducing the endogenous enzymes' ability to hydrolyze nutrients. Furthermore, mucous production is increased in high-fiber diets, which further impairs nutrient transport through the epithelial cells. The ability of xylanase to break down glycosidic bonds in the NSPs helps reduce digesta viscosity, provide nutrients in the form of free sugars, and minimize overall anti-nutritional effects of NSPs. The inclusion of soybean hulls allowed for reducing energy and increasing the fiber content of diets; this provided xylanase with a relatively challenging diet to express its potential. This setting represents an excellent example of what the current poultry industry is facing with the increased need to identify options that would allow for improved nutrient utilization of high-fiber ingredients to reduce cost. Added to that is the pressure of diverting high-energy ingredients to the ethanol production sector, leaving the poultry industry with a high-fiber variable byproduct ingredients to utilize.

### Environmental Pathogen Load

**Figure 1:**
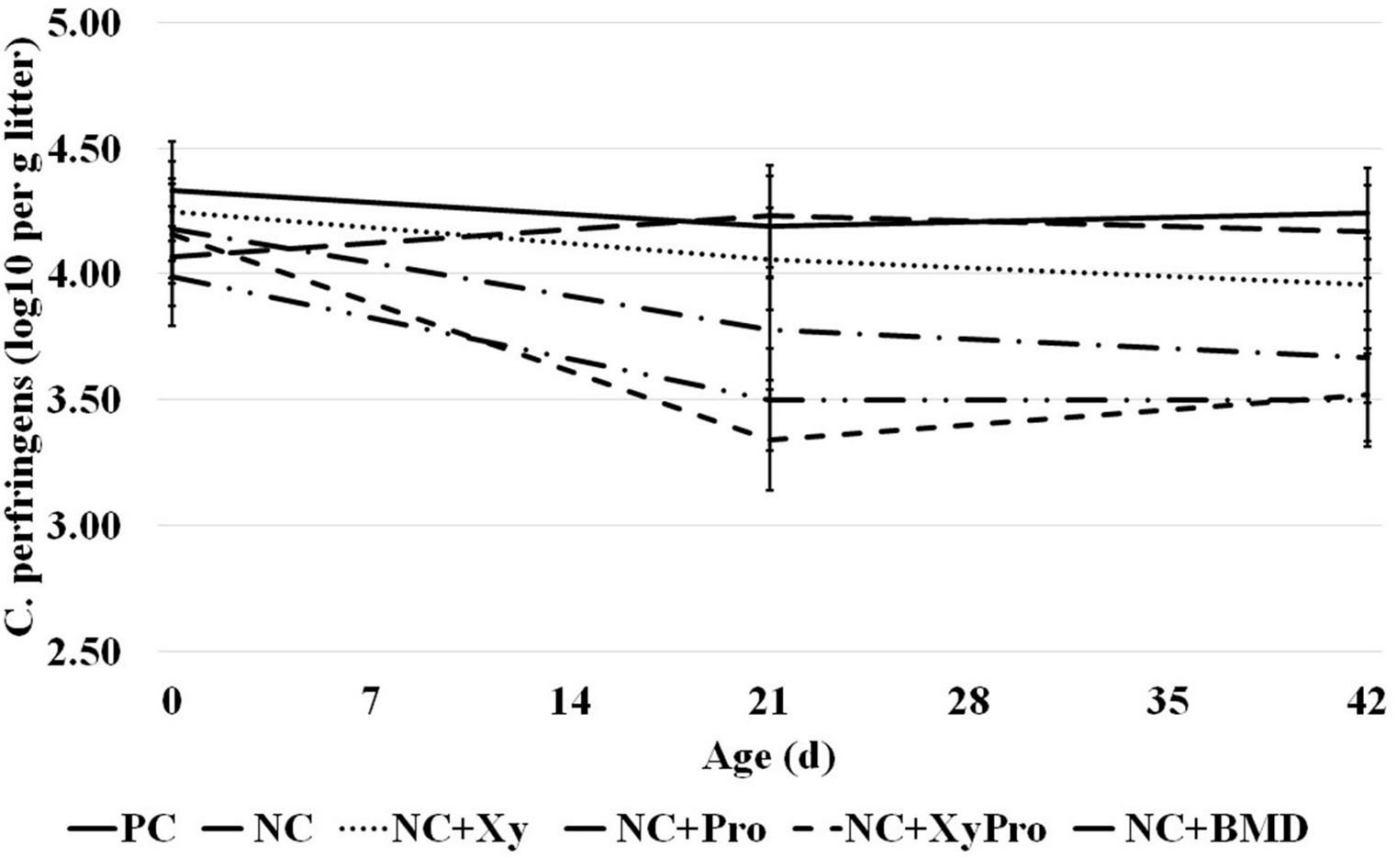
Abundance of *C. perfringens* in litter samples collected from pens with birds raised to 42 days and consumed one of the experimental diets: PC, positive control; NC, negative control with ME 130 kcal/kg lower than PC; NC + Xy, NC with xylanase; NC + Pro, NC with probiotics mix; NC + XyPro, NC with xylanase and probiotics mix; NC + BMD, NC with BMD (d0: *P* > 0.05; d21: *P* ≤ 0.05; d42: *P* ≤ 0.05).

The abundance of *C. perfringens* cells present in litter sampled prior to placement and 21 and 42 days was significantly reduced (*P* = 0.05) in pens from birds consuming diets supplemented with XyPro or BMD when compared to unsupplemented controls (Figure 1). Initial counts determined prior to placement had an average log_10_ of 4.12 ± 0.2 in all pens. A reduction by ~16% in litter *C. perfringens* cell count was achieved by either supplementation. The differences among treatments in *C. perfringens* load in the litter were not affected by energy level in the diet. Rather, the presence of either DFM or antibiotic had more profound effects on reducing the load. Oyeagu et al. (37) reported that beneficial bacterial counts increased in ileal and cecal contents of broilers when fed with diets supplemented with *Aspergillus* xylanase, which could probably be due to xylanase's ability to reduce digesta viscosity through the hydrolysis of the NSPs, which are also considered as the carbon source for intestinal microbiota.

In this study, both xylanase and DFM may have exerted part of their responses by changing the microbiota population; DFM is a competitive exclusion driver with xylanase facilitating a prebiotic effect and is a digesta viscosity-reducing agent. It has also been reported that several *Bacillus* strains, when used as DFM, have the ability to inhibit pathogens through the production of antimicrobials (38, 39). Furthermore, Park et al. (40) showed that broiler gut metabolites were altered by the *Bacillus* strain when used as DFM. They suggested that these metabolites might play a role in recognizing pathogen-associated molecular patterns (PAMPs) eventually leading to preventing intestinal disease and improving birds' growth. On the other hand, fermentation of soluble NSPs releases energy that can be utilized by the harmful bacteria harboring the gut leading to its propagation. Exogenous enzymes may lower total bacterial counts in intestinal contents, as already reported by several authors (41, 42), mainly gram positive cocci and enterobacteria (33, 34, 41), or populations of undesirable organisms such as *C. perfringens* (41), the cause of necrotic enteritis (43), as well as the thickening of the intestinal epithelium that may induce a reduction of nutrient absorption (44).

### Apparent Metabolizable Energy Digestibility

Reducing dietary ME in NC by 130 kcal/kg below ME of the PC resulted in a reduced digestibility of AMEn in the NC (Table 3). The improvement in AMEn digestibility compared to NC as a result of adding either xylanase or probiotic alone was intermediate. However, supplementing the NC with either XyPro or BMD improved AMEn by ~45 and 71 kcal at 21 days and by 48 and 70 kcal at 42 days, for XyPro and BMD, respectively. The inclusion of xylanase in monogastric diets reduces digesta viscosity and improves nutrient utilization by the degradation of NSPs into mono and oligosaccharides, which is reflected in improved nutrient digestibility of energy in this trial. Furthermore, researchers found that the probiotic effects of *B. subtilis* were more pronounced in nutrient-deficient broiler diets, resulting in improved energy digestibility (45).

**Table 3:** Least-square means for apparent metabolizable energy corrected for nitrogen (AMEn) of broilers raised to 42 days.

	**Treatment**						**SE**	***P*-value**
**Age (days)**	**PC**	**NC**	**NC + Xy**	**NC + Pro**	**NC + XyPro**	**NC + BMD**		
**AME (kcal/kg)**								
21	3,034^a^	2,889^e^	2,926^cd^	2,910^d^	2,934^c^	2,959^b^	6.856	0.0001
42	3,230^a^	3,078^e^	3,122^c^	3,102^d^	3,129^c^	3,151^b^	6.075	0.0001
**AMEn (kcal/kg)**								
21	3,004^a^	2,857^e^	2,895^cd^	2,879^d^	2,902^c^	2,928^b^	6.888	0.0001
42	3,202^a^	3,054^e^	3,095^c^	3,075^d^	3,102^c^	3,124^b^	6.091	0.0001

a−e *Means in a row within each variable with no common superscript differ significantly (P ≤ 0.05)*.

*PC, positive control; NC, negative control with ME 130 kcal/kg lower than PC; NC + Xy, NC with xylanase; NC + Pro, NC with probiotic; NC + XyPro, NC with xylanase and probiotic; NC + BMD, NC with BMD; SE, standard error for n = 8 pens*.

In conclusion, the current study's findings illustrate the potential for a synbiotic combination of xylanase enzyme (10 XU/g feed) with a DFM of *Bacillus* spp. (1 × 10^5^ CFU/g feed). This blend can be supplemented as a feed additive substituting AGP in broiler diets as well as compensating for a reduction up to 130 kcal ME/kg feed (~4.0% of energy content in a standard corn soy diet). This blend has proven its synergistic effect in improving live performance, reducing environmental microbial load, as well as improving energy utilization in birds raised to market weight. This blend could potentially allow for feeding diets with lower energy content, thus reducing production cost. Furthermore, energy reduction can be achieved by incorporating feed ingredients low in energy and high in fiber, which would also provide more substrate for the enzyme to utilize. Thus, this blend of xylanase and probiotic can be used as an alternative to antibiotic growth promoters in broiler diets even when formulated at ~4.0% less ME than a standard diet.

## Data Availability Statement

The datasets generated for this study are available on request to the corresponding authors.

## Ethics Statement

The animal study was reviewed and approved by Institutional Animal Care and Use Committee.

## Author Contributions

BN conceptualized and secured funding, designed the study and methodology, organized conducting the trial and collecting data, and performed data analysis and summary. The manuscript was written by BN and reviewed by both BN and J-JW. All authors have read and agreed to the published version of the manuscript.

## Conflict of Interest

J-JW was employed by company BioResource International Inc. The remaining author declares that the research was conducted in the absence of any commercial or financial relationships that could be construed as a potential conflict of interest.
